# HIV-1 capsid interacts with cytoskeletal-associated proteins for intracytoplasmic routing to the nucleus

**DOI:** 10.1186/1742-4690-10-S1-P34

**Published:** 2013-09-19

**Authors:** Juliette Fernandez, Kathleen Gärtner, Andreas Becker, Anne Danckaert, Sandie Munier, Anaba Zambo, Spencer Shorte, Yves Jacob, Pierre Charneau, Nathalie J Arhel

**Affiliations:** 1 Inserm U941, University Institute of Hematology, Saint-Louis Hospital, Paris, France; 2 Imagopole, Institut Pasteur, Paris, France; 3 Paris VII University, Institut Pasteur, CNRS UMR3569, Molecular Genetics of RNA Viruses Unit, Virology Department, Paris, France; 4 Molecular Virology and Vaccinology, Institut Pasteur, Paris, France

## Background

During infection, the Human Immunodeficiency Virus type 1 (HIV-1) uses the host cytoskeleton to traffic across the cytoplasm to the nucleus where its genome integrates into the host DNA. We have previously shown that HIV retrograde transport (i.e. towards the nucleus) results from the successive transfers from fast microtubule-directed movement to slower actin-mediated movement closer to the nuclear compartment, resulting in docking at the nuclear pore [1]. However, neither the cellular cytoplasmic component(s) nor the viral protein(s) that interact to mediate transport have yet been identified. Recent data show that HIV-1 uncoating does not occur immediately after cell entry but near the nuclear membrane, suggesting that the viral structure interacting with the cytoskeleton during early retrograde trafficking might be the capsid.

## Materials and methods

Based on this hypothesis, we carried out a yeast-two-hybrid screen using HIV-1 monomeric capsid protein (p24) as bait. We then characterised the identified proteins for their ability to associate with assembled capsids and their effect on infectivity, trafficking, and nuclear import.

## Results

Our yeast-two-hybrid assay identified 34 new putative interaction partners for HIV p24, including four cytoskeletal components. Interaction with assembled HIV capsids was confirmed for two of the four cytoskeletal proteins. Their depletion using RNA interference led to a profound reduction in HIV-1 infectivity in single cycle infection assays, pointing to a key role in the early steps of HIV-1 replication. Furthermore, confocal microscopy revealed a characteristic accumulation of HIV-1 capsids away from the nuclear membrane and an overall defect in nuclear import.

## Conclusion

This work identifies assembled HIV-1 capsid as the viral determinant of transport to the nucleus and two cytoskeletal proteins as new HIV capsid interaction partners that mediate viral retrograde transport.
